# Supplementary material for: Adversarial confound regression and uncertainty measurements to classify heterogeneous clinical MRI in Mass General Brigham
Source: PLoS One. 2023 Mar 2;18(3):e0277572. doi: 10.1371/journal.pone.0277572 (PMC9980829; doi:10.1371/journal.pone.0277572)
Supplement: S1 File — (PDF) [file pone.0277572.s001.pdf]

Training set sites

Table S1. Sites for the training set (MGH Pre-2019), as well as the numbers of AD and Controls scanned at each

MRI Site Name	No. AD	No. CTRL	Norm AD (No. AD / Total AD)	Norm CTRL (No. CTRL / Total CTRL)	Ratio (Norm AD : Norm CTRL)
MR1WA	275	370	0.0653	0.0293	2.2243
ERMR	79	294	0.0188	0.0233	0.8041
LI_MR	135	779	0.0320	0.0618	0.5186
LI	958	5468	0.2274	0.4337	0.5243
MR3_ELL2	12	83	0.0028	0.0066	0.4327
MR1CH	196	245	0.0465	0.0194	2.3941
MR3EL2	171	472	0.0406	0.0374	1.0842
MR1W1	246	566	0.0584	0.0449	1.3007
PR62ELL2	34	129	0.0081	0.0102	0.7888
MRC40168	228	294	0.0541	0.0233	2.3208
MR1A2	142	273	0.0337	0.0217	1.5566
PR67ELL2	52	162	0.0123	0.0128	0.9606
MRFND1	228	715	0.0541	0.0567	0.9543
MR1L6	196	98	0.0465	0.0078	5.9853
MRS1OW	74	286	0.0176	0.0227	0.7743
MR2WA	89	98	0.0211	0.0078	2.7178
MR2EL2	94	238	0.0223	0.0189	1.1820
EXHDMRI	21	27	0.0050	0.0021	2.3276
MR1Y6	124	179	0.0294	0.0142	2.0731
MR2CH	149	187	0.0354	0.0148	2.3845
PR66ELL2	36	252	0.0085	0.0200	0.4275
MR1NS	161	192	0.0382	0.0152	2.5095
PR248	44	181	0.0104	0.0144	0.7275
MRC25157	44	64	0.0104	0.0051	2.0574
MR2Y6	160	284	0.0380	0.0225	1.6860
MR1NY	2	12	0.0005	0.0010	0.4988
MR1EL2	131	275	0.0311	0.0218	1.4256
CHCMRI	57	156	0.0135	0.0124	1.0935
MR3Y6	1	3	0.0002	0.0002	0.9975
MRC20597	11	52	0.0026	0.0041	0.6331
MRC35022	28	95	0.0066	0.0075	0.8820
CHCMR2	4	15	0.0009	0.0012	0.7980
GEMS	0	5	0.0000	0.0004	0.0000
MEDPC	9	12	0.0021	0.0010	2.2445
M247OC0	0	1	0.0000	0.0001	0.0000
BAY4OC	2	2	0.0005	0.0002	2.9926
BAY1OW0	0	1	0.0000	0.0001	0.0000
MR3CH	11	16	0.0026	0.0013	2.0574
BAY3OC	0	8	0.0000	0.0006	0.0000
MR3WA	8	16	0.0019	0.0013	1.4963
MRS3OC0	1	2	0.0002	0.0002	1.4963
MRS5MRS5	0	1	0.0000	0.0001	0.0000
**TOTAL**	**4213**	**12608**	**1.0000**	**1.0000**	

Training set sample images

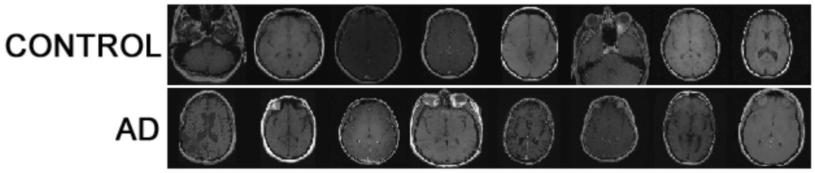

Fig S1. Sample images. Slices of random AD and control MRIs from the dataset, showing the general heterogeneity of the images.
